# Breast cancer screening coverage is severely reduced among women who reside in segregated areas: a cross-sectional investigation in Hungary

**DOI:** 10.3389/fpubh.2025.1500098

**Published:** 2025-07-14

**Authors:** Aseel Odeh, Kasabji Feras, Ferenc Vincze, Kinga Lakatos, Anita Pálinkás, László Kőrösi, László Ulicska, Karolina Kósa, János Sándor

**Affiliations:** ^1^Department of Public Health and Epidemiology, Faculty of Medicine, University of Debrecen, Debrecen, Hungary; ^2^HUN-REN-UD Public Health Research Group, Department of Public Health and Epidemiology, Faculty of Medicine, University of Debrecen, Debrecen, Hungary; ^3^Doctoral School of Health Sciences, University of Debrecen, Debrecen, Hungary; ^4^National Health Insurance Fund, Budapest, Hungary; ^5^Deputy State Secretariat for Social Inclusion, Ministry of Interior, Budapest, Hungary; ^6^Department of Behavioral Sciences, Faculty of Medicine, University of Debrecen, Debrecen, Hungary

**Keywords:** residential segregation, breast cancer screening, cross-sectional study, inequality, monitoring

## Abstract

**Background:**

This study investigated disparities in breast cancer screening participation between living in residential segregations (SAs, segregated areas defined by clustering of low levels of income and education) and in non-segregated, complementary areas (CAs) of Hungary.

**Methods:**

In a nationwide cross-sectional study, data from 2019 were obtained from the National Health Insurance Fund (NHIF). In accordance with the Hungarian recommendation, the target group was composed of women aged 45–65, and screening participation was evaluated as appropriate if the women participated in mammography within 2 years. Standardized participation ratios (sPRs) were calculated for each SA and CA. These ratios were adjusted for age and eligibility for exemption certificates. The calculations were done for each general medical practice (GMP) serving a population with at least one SA, as well as for the whole country. The level of inequality was quantified by the relative standardized participation ratio (rsPR) by comparing sPR in the segregated versus non-segregated areas.

**Results:**

The study identified 11,581 observed breast cancer screening cases in SAs, compared with 417,891 in CAs, with target populations of 45,185 in SAs and 984,198 in CAs. In general, crude participation rates were significantly lower in SAs (25.6%) than in CAs (42.5%), with a rsPR of 0.62 (95% CI: 0.61–0.63). The impact of segregation on national screening coverage was negligible (population attributable risk: −1.2%). The GMP-level rsPR varied widely with a median of 0.653 and interquartile range (IQR) of 0.464–0.867. Notably, 15.6% of the GMPs had significantly reduced rsPR.

**Conclusion:**

This study demonstrated that breast cancer screening coverage is considerably lower among women living in SAs than in those living in non-segregated areas. GMPs showed substantial variability with respect to segregation related inequality. There was a remarkable proportion of GMPs without local inequality. The impact of segregation on national breast cancer screening participation was negligible. According to our observations, the segregation-specific indicators should be included in screening monitoring, and its results should be feedback to local authorities and stakeholders in order to identify and address local problems of screening organization to reduce inequalities.

## Introduction

Breast cancer has become the most common cancer among women worldwide. Moreover, a 31% increase in the incidence of female breast cancer is predicted to occur from 2020 to 2040 (1–3). Health loss, in both DALYs and deaths, has been increasing globally as well, apart from high-income countries, where health loss has steadily decreased over the last three decades (4, 5). Although our knowledge of the etiology of breast cancer is increasing, primary prevention of breast cancer faces challenges due to the complex interplay of genetic, environmental, and lifestyle factors (6).

Given these limitations, properly organized screening with improved treatment mainly determines the effectiveness of breast cancer control (7). Compared with opportunistic screening, organized screening is more effective at reducing cancer mortality (8). Recent data show varying participation rates for breast cancer screening in different populations. In the United States, the rate was 76% among women aged 50–74 years. In European regions, it ranged from 49 to 69%. By 2019, in Europe, organized mammography screening has demonstrated significant success and potential for further development, preventing 21,680 deaths but failing to prevent 12,434 deaths due to incomplete screening coverage (9, 10).

Observations from diverse countries and regions suggest that individuals with low socioeconomic status (SES) encounter substantial barriers in regard to accessing breast cancer screening services. Reduced income, lower educational achievement, and residing in rural areas have been recognized as strong indicators predicting decreased participation in breast cancer screening services (11–15). Additionally, in the U. S., racial/ethnic minority women reported common logistical, psychological, cultural, and social barriers; however, the primary barrier for certain population groups was the lack of medical insurance (16–21). Some studies in the U. S., where different measures are used to indicate residential segregation, have demonstrated the hindering role of segregation in breast cancer screening coverage (22–25), whereas others have not (26–29). In Europe, most but not all studies, regardless of the socioeconomic indicators used, show that area-level deprivation is linked to lower breast cancer screening rates. In France, studies found that women in deprived neighborhoods were significantly less likely to participate in screening than those in affluent areas (30, 31). However, another study from France found that screening was highest in moderately deprived areas (32). In England, while there is generally a negative association between deprivation and screening uptake, some London areas show higher participation rates in deprived regions (33–37). This pattern is also observed in Sweden, where area-level deprivation impacts screening attendance, though the relationship is complex according to the detailed analysis. While certain disadvantageous demographic factors are associated with lower attendance, some areas facing these challenges still exhibit high participation rates (38).

In 2001, when breast cancer screening was opportunistic in Hungary, the frequency of mammography in the primary target population for breast cancer screening (females aged 45–64) was 17.5% a year. At that time, the higher mammography rates were closely tied to advantageous socioeconomic characteristics such as a lower unemployment rate, better income, and a higher level of education at the district level. By 2005, with the introduction of organized screening services (in which all the women belong to the target group are invited for a free-of-charge breast cancer screening ensuring them an appointment at the nearest screening center), these socioeconomic factors had lost their significance, and this change was accompanied by an increase in the participation rate to 29.4% a year (39). Despite this achievement in Hungary, the average participation rate across Europe was 53.4%, ranging from 19.4 to 88.9%, leaving Hungarian screening below the European reference (40). Hungarian breast cancer screening prevented only 318 deaths in 2018, whereas 481 was the expected number of prevented cases on the basis of the European reference (9).

Furthermore, according to individual level investigations, targeted health promotion initiatives remain essential to increase participation, especially among women with lower education, perceived income, and health care utilization (40, 41). Notably, Roma people, who are among the most disadvantaged groups with critical health status in the country, face significant barriers to accessing quality health care services (42, 43). Although the lack of ethnicity specific data prevents the quantification of poor screening implementation among Roma women, the restricted use of secondary outpatient services in general is well demonstrated for this minority (44). Approximately 4% of the Hungarian population resides in marginalized areas within settlement segregates. Roma is seriously overrepresented in these communities. This population is likely the most vulnerable population of the country (45).

## Objectives

This study (1) examined breast cancer screening participation rates among residents of segregated areas and (2) compared them to those among residents of non-segregated areas of the same settlements in order to explore whether the segregation related disparity is prevented by the population based screening organization or not in Hungary. (3) By assessing the participation inequality related to segregation, this research aimed to determine whether Hungarian segregated areas as a whole or only a part of the segregated areas should be considered as target group that require distinct attention to improve screening participation.

## Methods

### Mapping of the segregated areas

To specify marginalized and disadvantaged communities to be targeted by interventions, a governmental decree has defined socially disadvantaged clusters in Hungary as clusters of residents aged 18–59 within settlements (towns and villages), characterized by a lack of higher than primary level education and a lack of work-related income (segregated areas, SAs). The Hungarian Central Statistical Office identifies these clusters and their complementary (non-segregated) areas (CAs) of settlements across all Hungarian settlements. The place of each household is classified as located in either SA or CA in a mutually exclusive manner. By utilizing the output of this system and possessing the addresses of adults, the National Health Insurance Fund (NHIF) can discern clients residing in SAs or CAs. Consequently, all Hungarian adults can be classified as inhabitants of an SA or a CA.

### Setting

We conducted a comprehensive nationwide cross-sectional analysis at the end of 2019 focusing on populations served by general medical practices (GMPs). The study population was composed of women aged 45–65 years; the age group of women targeted for breast cancer screening every 2 years. Out of 4,851 Hungarian GMPs 3,557 provided care for women aged 45–65 living in SAs.

### Data sources

All the data were provided by the NHIF, the single provider of health insurance for the Hungarian population, which contracts with each Hungarian GMP. The NHIF produces an indicator of the breast cancer screening participation rate for each GMP as a part of its routine primary care monitoring system from 2009.

The number of women aged 45–65 cared for by GMPs was provided by the NHIF. The target population of each GMP was divided into segregated (N_SA_) and non-segregated (N_CA_) parts. The number of women who participated in mammography within 24 months (in 2018 or 2019) belonging to the breast cancer screening target population was also provided by the NHIF for GMPs specified for SA (M_SA_) and CA (M_CA_). Both the target population and case numbers were ensured by age groups (5-year bands) and by eligibility for the exemption certificate (deprived patients with at least one chronic illness are supported by an exemption certificate that is released by the local municipality on the basis of the recommendation of the patient’s GP).

### Statistical analysis

SA and CA specific crude participation ratios (cPRs) were computed for each GMP serving a population with at least one SA. Furthermore, stratum-specific national cPR values were used for indirect standardization. Age and eligibility for exemption certificate standardized participation ratios (sPRs) were computed for each SA and CA along with corresponding 95% confidence intervals (95% CIs). The relative standardized participation ratio (rsPR) was calculated as the ratio of SA specific sPR and CA specific sPR for each GMP. All the risk measures were aggregated for the whole study population. Additionally, the number of excess cases in SAs and the attributable risks for screening participation were determined for SAs, for the studied population, and for the entire population of the country.

In order to identify GMPs where the sPR_SA_ and sPR_CA_ differed significantly, the expected number of cases in SA was corrected with the sPR_CA_ computed for the same GMP. In this manner, the locally adjusted standardized participation ratios (lsPR) were calculated for each SA. The difference between the number of SA specific observed and SA specific locally corrected expected cases were tested by mid-p test (46). Using these test results, GMPs where the screening coverage was significantly less in the SA than in the CA were distinguished from GMPs where the SA and the CA specific measures were not differentiated from each other, and from the GMPs where the screening coverage was significantly higher in the SA than in the CA.

Data were analyzed using SPSS version 20 (IBM Corporation, New York, NY, USA).

### Ethical permission

This study was a secondary data analysis that utilized data aggregated for CAs and SAs. There were no person level data used for the statistical evaluation. Therefore, written informed consent was not required from the patients.

The person level data were processed by the NHIF, which is empowered for that task by law. The process of producing aggregated indicators for SAs and CAs for further analyzes within the secret internal data handling system of the NHIF was approved by the Office of the Commissioner for Fundamental Rights (AJB-3147/2013), the general director of the NHIF (E0101/215-3/2014), and the Hungarian National Authority for Data Protection and Freedom of Information (NAIH/2015/826/7N).

## Results

**Figure 1:**
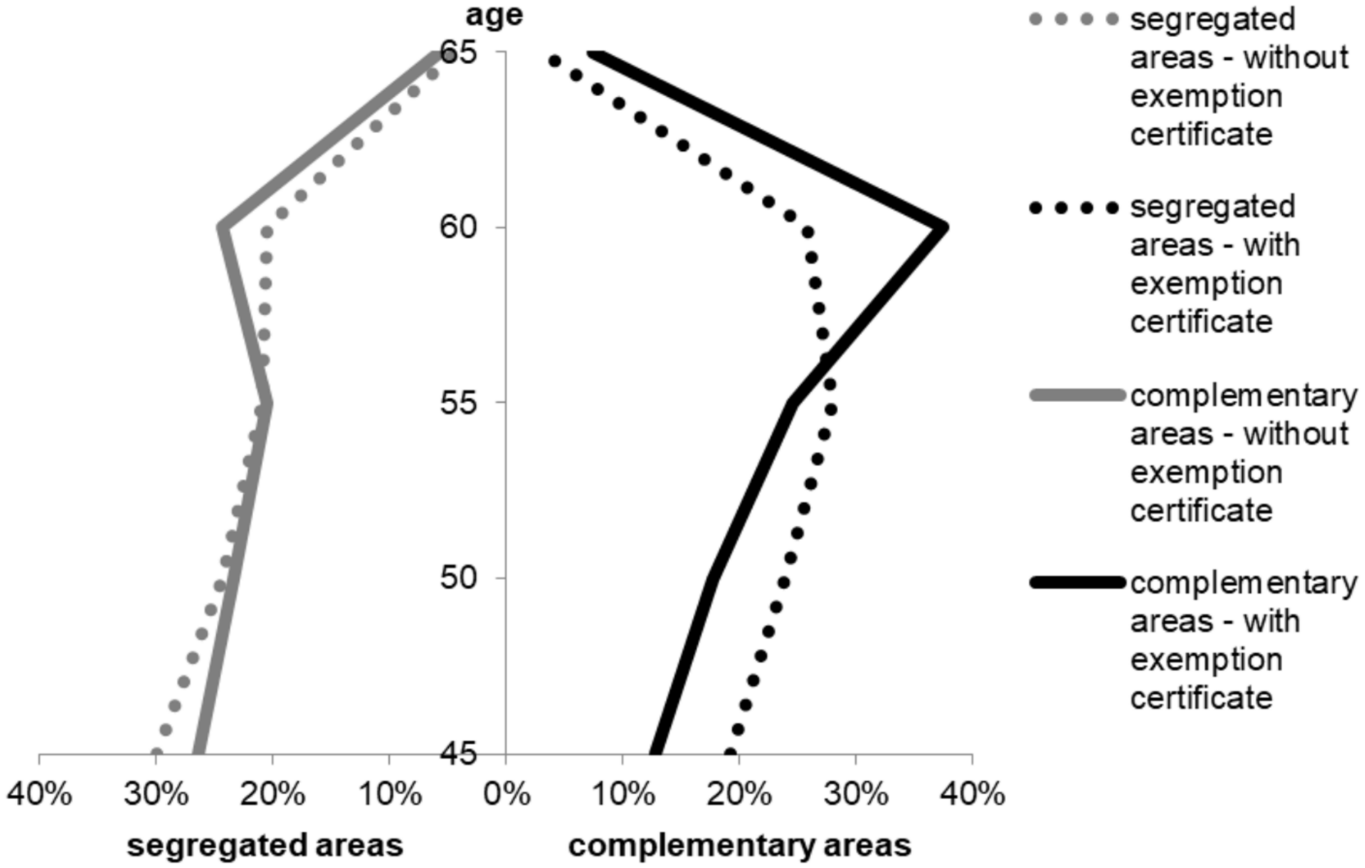
Demographic structure of the target populations of breast cancer screenings in segregated and complementary areas of GMPs.

The age distribution shifted toward being younger in SA than in CA. The proportion of women eligible for exemption certificates was much greater in SA (n = 7,138; 15.8%) than in CA (n = 33,671; 3.4%). A total of 4,851 GMPs were evaluated (Figure 1).

**Figure 2:**
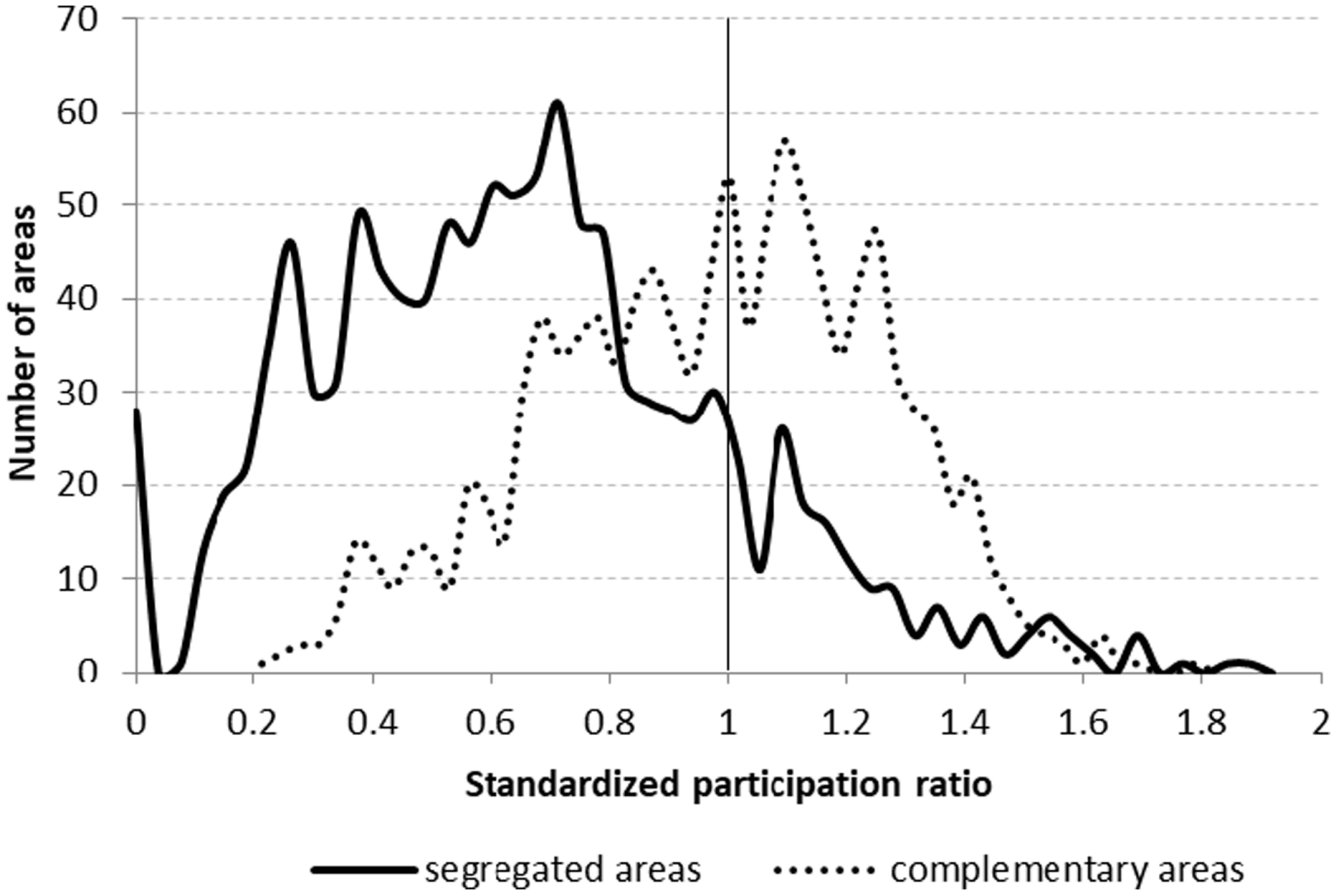
Histograms of age and exemption certificate eligibility standardized participation ratios for breast cancer screening among women aged 45–65 years residing in segregated areas and in complementary areas in Hungary.

The cPR for the whole country was 42.1% (572,269 women among 1,357,923 women who had participated in breast cancer screening within 24 months). The target population in SAs was 45,185, and 984,198 in CAs. The number of women who participated in screening was 11,581 and 417,891 in the SAs and CAs, respectively. The overall cPR was 25.6% for SAs and 42.5% for CAs. The standardized participation ratio in SAs (sPR_SA_ = 0.627; 95% CI: 0.616–0.639) was significantly lower than that in CAs (sPR_CA_ = 1.007; 95% CI: 1.004–1.010) (Table 1). The GMP-level sPRs varied widely for both SAs (median: 0.610, IQR: 0.452) and CAs (median: 0.985, IQR: 0.414). The difference was significant according to the Mann–Whitney test (*p* < 0.001) (Figure 2).

**Table 1:** Risk measures for participation in breast cancer screening within 24 months among 45–65 year old women in Hungary living in segregated or complementary areas.

Risk measures	Segregated areas of the country (a)	Complementary areas of the whole country (b)	Complementary areas belong to GMPs providing segregated area (c)	Study population (target population in GMPs with at least 1 segregated area) (a + c)	Whole country (a + b)
Target population	45,185	1,312,738	984,198	1,029,383	1,357,923
Observed cases	11,581	560,688	417,891	429,472	572,269
Crude participation ratio	25.6%	42.7%	42.5%	41.7%	42.1%
Expected number of cases	18,471	553,797	414,984	433,455	572,269
Standardized participation ratio*	0.627 [0.616–0.639]	1.012 [1.010–1.015]	1.007 [1.004–1.010]	0.991 [0.988–0.994]	1.000 [0.997–1.003]
Number of excess cases	−6,890		–	–	
Attributable risk	−59.5%	–	–	−1.6%	−1.2%

^*^with 95% confidence interval.

**Figure 3:**
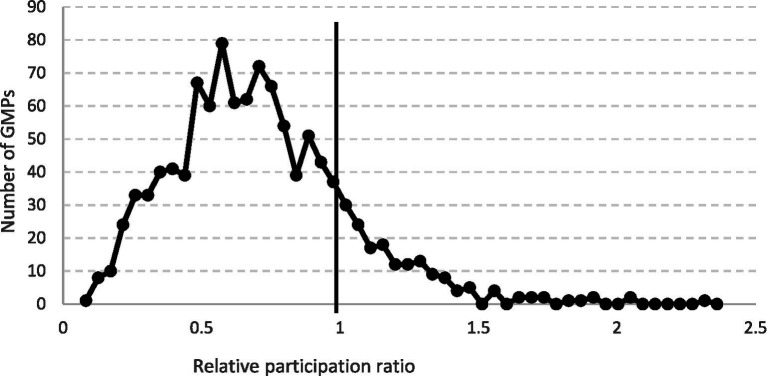
Relative participation ratio in breast cancer screening among women aged 45–65 years in segregated areas in comparison to that in complementary areas by general medical practices (GMPs) in Hungary.

The relative participation of women living in SAs was significantly lower than that of women living in CAs (rsPR = 0.623, 95% CI: 0.611–0.634). The distribution of the rsPR of GMPs covered a wide range (median = 0.653, IQR: 0.464–0.867) (Figure 3).

A total of 190 (5.34%) of the GMPs had significantly reduced rsPR. In these GMPs, the excess number of cases was 2613, that is there were 2,613 women whose missed screening can be attributed to their residential place in a segregates and to a GMP level factor, which reduces the effectiveness of screening organizations. Meanwhile, in these GMPs, the number of excess cases, the number of implemented screenings in non-segregated areas was 1,271 higher than expected on the basis of the Hungarian references (Table 2).

**Table 2:** Screening participation according to the relative mammography coverage in the segregated areas (SA) compared to the complementary areas (CA) part of the population provided by a GMP.

Risk measures		Relative coverage in SA			Total
		Significantly lower	Not deviated from the CA	Significantly higher	
Number of GMPs		190 (5.34%)	3,363 (94.54%)	4 (0.11%)	3,557
SA	Observed cases	1997	9,538	46	11,581
	Expected number of cases*	4,610	13,809	52	18,471
	Excess number of cases	−2,613	−4,271	−6	−6,891
	Standardized participation ratio**	0.433 [0.415–0.453]	0.691 [0.677–0.705]	0.887 [0.665–1.185]	0.627 [0.616–0.638]
CA	Observed cases	19,084	398,702	105	417,891
	Expected number of cases*	17,813	396,862	309	414,984
	Excess number of cases	1,271	1840	−204	2,907
	Standardized participation ratio**	1.071 [1.056–1.087]	1.005 [1.002–1.008]	0.34 [0.281–0.412]	1.007 [1.004–1.01]

^*^calculated by the national reference values.

^**^with 95% confidence interval.

**Figure 4:**
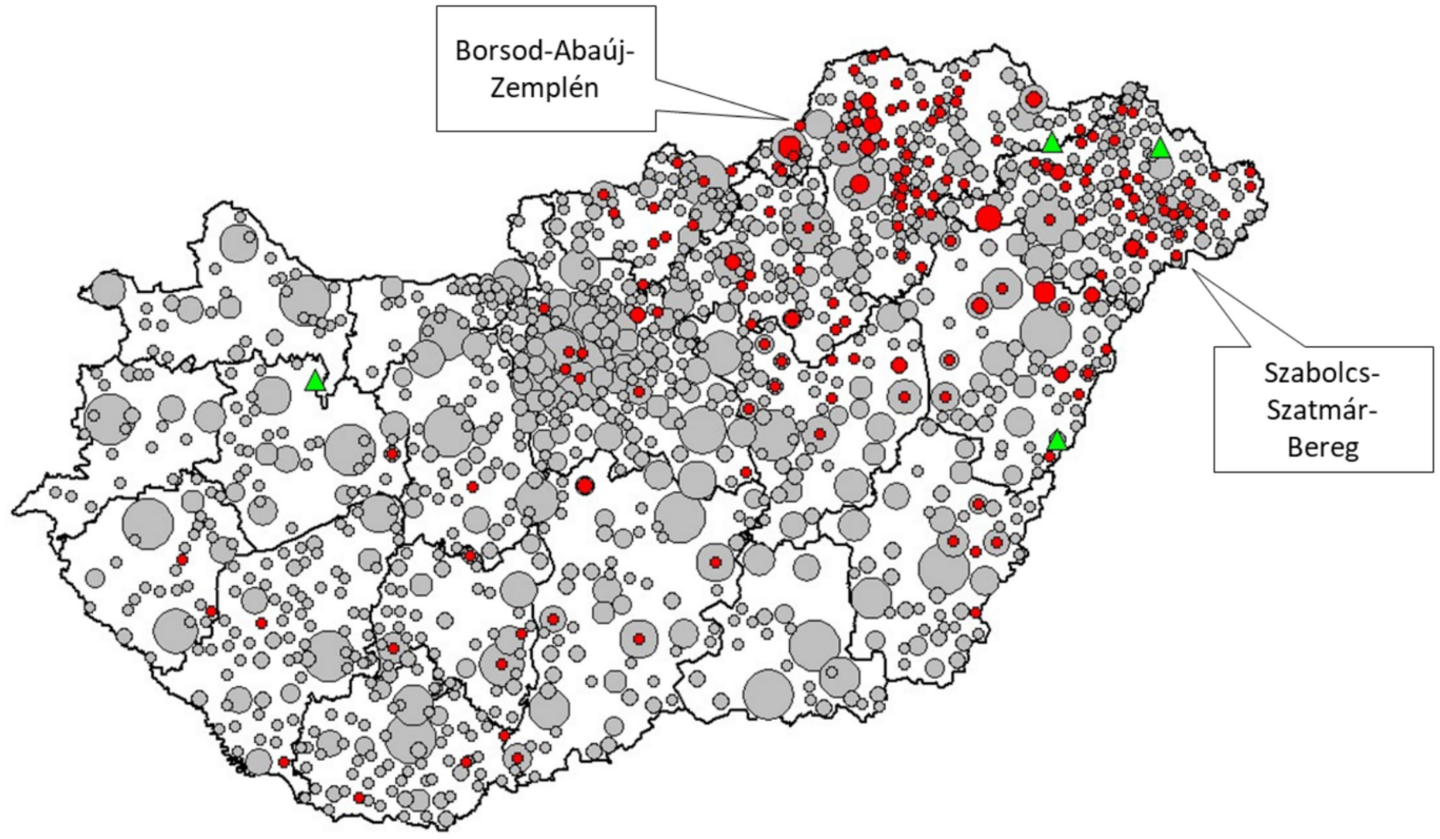
Relative standardized participation ratio in mammography screening among 45–65-year-old women living in areas segregated by general medical practices (GMPs) in Hungarian counties. (red: standardized participation ratio in the segregated area is significantly less than that in the complementary area; gray: standardized participation ratio in the segregated area does not deviate significantly from that in the complementary area; green: standardized participation ratio in the segregated area is significantly greater than that in the complementary area; size of red and grey symbols is proportional to the number of GMPs with the same statistical evaluation in a settlement, each green triangle corresponds to one GMP).

A high degree of spatial concentration was observed in the distribution of GMPs’ rsPR (Figure 4). Two counties (Borsod-Abaúj-Zemplén: 17.56, 95% CI: 13.64–22.06%; Szabolcs-Szatmár-Bereg: 17.32, 95% CI: 12.88–22.55%) had a significantly greater proportion of vulnerable GMPs with significantly reduced rsPR (Details of the county specific observations in the Appendix).

The number of completed breast cancer screenings in the SAs was 6,890 less than expected. The corresponding etiological fraction in the SAs was −59.5%, and the population attributable risk for the investigated population was −1.6%. Considering the whole population of the country, the population attributable risk was −1.2% (Table 1).

## Discussion

### Main findings

Our research in Hungary reveals that segregated areas have lower rates of mammography participation than that of non-segregated areas. This finding is consistent with the main observations in other developed countries concerning the adverse effects of socioeconomic deprivation on the use of breast cancer screening (47–50). Although, this observed difference between SAs and CAs is not extreme compared to observations from the US (28) and Sweden (38), the screening rate of 25.6% observed in SAs is much less than the reported screening rate in less developed countries such as Turkey (32.2%), China (54.8%), and Brazil (44–63% by regions). The reported rate from Iran (12.3%) and Nigerian health care workers (15.4%) were under the observed SA rate (51–55).

Nevertheless, owing to the very small population attributable risk (−1.2%), the influence of segregation on Hungary’s overall low mammography screening rates appears to be not significant, implying that improving the effectiveness of screening in SAs could not remarkably increase the low mammography coverage in the country.

Although segregation focused interventions cannot improve the poor within-European position of Hungary (56), the considerable attributable risk of −59.5% in SAs urges local interventions. Considering the substantial variability in the GMP-level utilization of mammography services, benchmarking could be a viable approach to identify and implement good practices.

### Strengths and limitations

This study covered the entire country, eliminating selection bias and ensuring proper statistical power. Strict definitions for segregation, as provided by the government, and for health care use data, as defined by the NHIF, prevented misclassification.

This study used administrative registration of the residential addresses. Discrepancies between registered and actual residential addresses could introduce bias.

The GMP level statistical testing was highly conservative due to the correction of the mid-p testing, and it had also small statistical power due to the relatively small number of cases. These circumstances could explain the apparent discrepancy between the small number of the GMPs with significant difference between SA and CA specific screening coverages, and the huge difference of the summarized coverages of SAs and CAs.

Additionally, SAs were underparameterized. Although age and SES (indicated by eligibility for exemption certificates) were controlled by standardization, further investigations are needed to identify factors responsible for the heterogeneous screening rates of SAs. These additional investigations with more extended set of confounding factors could contribute to the elaboration of the appropriate methodology of remedial interventions.

### Implications

Although the importance of socioeconomic factors in terms of the screening participation rate is indisputable, their effects do not necessarily prevail. Experiences from European countries (57) and from the United States (26–29), and observations from our study demonstrated that the transformation of socioeconomic inequalities into screening inequality can be prevented.

Many mechanisms of this transformation are well known: cultural factors, such as traditional roles within segregated areas that prioritize their roles as daughters, wives, or mothers over their own health needs (58); embarrassment due to nudity (59, 60); distrust of the health care system (61); decreased accessibility of health care (14); and improper health literacy (62). Tailored interventions accounting for these factors, such as community outreach programs in segregated areas, education campaigns regarding patient privacy, mobile screening units to improve accessibility (63, 64), financial incentives (transportation vouchers, childcare assistance, monetary rewards) (65), and the involvement of GPs in monitoring breast cancer screening participation by checking proper participation in screening in the case of GP visits (65, 66), can prevent the generation of disparity.

Because, there are many settlements in Hungary without significant difference between SA and CA screening rates, there is many local experiences in preventing segregation elicited inequality. If the screening participation monitoring could use SA and CA level indicators, local good practices could be identified, and the elaboration of local interventions could be supported. Adequate monitoring and proper interaction with local actors could improve the involvement of local resources in problem management, as well.

## Conclusion

According to our study, breast cancer screening coverage is considerably lower in the population living in segregated areas of settlements than in the population living in nonsegregated areas of settlements. GMPs showed considerable variability with respect to segregation-related inequality in breast cancer screening but a considerable proportion of GMPs had no local inequality in this regard. The impact of segregation on country-level breast cancer screening participation was negligible.

According to our observations, the segregation-specific indicators should be included in screening monitoring, and its results should be feedback to local authorities and stakeholders in order to identify and address local problems of screening organization to reduce inequalities.

## Data Availability

The data analyzed in this study is subject to the following licenses/restrictions: the datasets used and/or analyzed during the current study are available from the corresponding author on reasonable request. Requests to access these datasets should be directed to János Sándor, janos.sandor@med.unideb.hu.

## References

[1] Wu Z Liu Y Li X Song B Ni C Lin F. Factors associated with breast cancer screening participation among women in mainland China: a systematic review. BMJ Open. (2019) 9:e028705. doi: 10.1136/bmjopen-2018-028705, PMID: 31455705 PMC6720337

[2] Interactive data visuals. (2022) Available online at: https://www.healthdata.org/data-tools-practices/interactive-data-visuals. Accessed 12 Aug 2024.

[3] Bray F Laversanne M Sung H Ferlay J Siegel RL Soerjomataram I. Global cancer statistics 2022: GLOBOCAN estimates of incidence and mortality worldwide for 36 cancers in 185 countries. CA Cancer J Clin. (2024) 74:229–63. doi: 10.3322/caac.21834, PMID: 38572751

[4] Wojtyla C Bertuccio P Ciebiera M La Vecchia C. Breast Cancer mortality in the Americas and Australasia over the period 1980-2017 with predictions for 2025. Biology. (2021) 10:814. doi: 10.3390/biology10080814, PMID: 34440046 PMC8389642

[5] Ferlay J Partensky C Bray F. More deaths from pancreatic cancer than breast cancer in the EU by 2017. Acta Oncol Stockh Swed. (2016) 55:1158–60. doi: 10.1080/0284186X.2016.1197419, PMID: 27551890

[6] Sun Y-S Zhao Z Yang Z-N Xu F Lu H-J Zhu Z-Y. Risk factors and preventions of breast Cancer. Int J Biol Sci. (2017) 13:1387–97. doi: 10.7150/ijbs.21635, PMID: 29209143 PMC5715522

[7] Kolak A Kamińska M Sygit K Budny A Surdyka D Kukiełka-Budny B. Primary and secondary prevention of breast cancer. Ann Agric Environ Med AAEM. (2017) 24:549–53. doi: 10.26444/aaem/75943, PMID: 29284222

[8] Miles A Cockburn J Smith RA Wardle J. A perspective from countries using organized screening programs. Cancer. (2004) 101:1201–13. doi: 10.1002/cncr.20505, PMID: 15316915

[9] Zielonke N Kregting LM Heijnsdijk EAM Veerus P Heinävaara S McKee M. The potential of breast cancer screening in Europe. Int J Cancer. (2021) 148:406–18. doi: 10.1002/ijc.33204, PMID: 32683673 PMC7754503

[10] Zielonke N Gini A Jansen EEL Anttila A Segnan N Ponti A. Evidence for reducing cancer-specific mortality due to screening for breast cancer in Europe: a systematic review. Eur J Cancer Oxf Engl. (1990) 127:191–206. doi: 10.1016/j.ejca.2019.12.010, PMID: 31932175

[11] Donnelly TT Al Khater A-H Al Kuwari MG Al-Bader SB Al-Meer N Abdulmalik M. Do socioeconomic factors influence breast cancer screening practices among Arab women in Qatar? BMJ Open. (2015) 5:e005596. doi: 10.1136/bmjopen-2014-005596, PMID: 25613951 PMC4305075

[12] Akinyemiju T Ogunsina K Sakhuja S Ogbhodo V Braithwaite D. Life-course socioeconomic status and breast and cervical cancer screening: analysis of the WHO’S study on global ageing and adult health (SAGE). BMJ Open. (2016) 6:e012753. doi: 10.1136/bmjopen-2016-012753, PMID: 27881528 PMC5129035

[13] Nuche-Berenguer B Sakellariou D. Socioeconomic determinants of cancer screening utilisation in Latin America: a systematic review. Plo S One. (2019) 14:e0225667. doi: 10.1371/journal.pone.0225667, PMID: 31765426 PMC6876872

[14] Özkan İ Taylan S. Barriers to women’s breast cancer screening behaviors in several countries: a meta-synthesis study. Health Care Women Int. (2021) 42:1013–43. doi: 10.1080/07399332.2020.1814777, PMID: 32877315

[15] Al Rifai R Nakamura K. Differences in breast and cervical Cancer screening rates in Jordan among women from different socioeconomic strata: analysis of the 2012 population-based household survey. Asian Pac J Cancer Prev APJCP. (2015) 16:6697–704. doi: 10.7314/APJCP.2015.16.15.6697, PMID: 26434897

[16] Smith D Thomson K Bambra C Todd A. The breast cancer paradox: a systematic review of the association between area-level deprivation and breast cancer screening uptake in Europe. Cancer Epidemiol. (2019) 60:77–85. doi: 10.1016/j.canep.2019.03.008, PMID: 30927689 PMC6547165

[17] Miller BC Bowers JM Payne JB Moyer A. Barriers to mammography screening among racial and ethnic minority women. Soc Sci Med. (1982) 239:112494. doi: 10.1016/j.socscimed.2019.112494, PMID: 31513931

[18] Adunlin G Cyrus JW Asare M Sabik LM. Barriers and facilitators to breast and cervical Cancer screening among immigrants in the United States. J Immigr Minor Health. (2019) 21:606–58. doi: 10.1007/s10903-018-0794-6, PMID: 30117005

[19] Oh KM Taylor KL Jacobsen KH. Breast Cancer screening among Korean Americans: a systematic review. J Community Health. (2017) 42:324–32. doi: 10.1007/s10900-016-0258-7, PMID: 27678390

[20] Anderson de Cuevas RM Saini P Roberts D Beaver K Chandrashekar M Jain A. A systematic review of barriers and enablers to south Asian women’s attendance for asymptomatic screening of breast and cervical cancers in emigrant countries. BMJ Open. (2018) 8:e020892. doi: 10.1136/bmjopen-2017-020892, PMID: 29982210 PMC6042536

[21] Tran L Tran P. US urban-rural disparities in breast cancer-screening practices at the national, regional, and state level, 2012-2016. Cancer Causes Control CCC. (2019) 30:1045–55. doi: 10.1007/s10552-019-01217-8, PMID: 31428890

[22] Dai D. Black residential segregation, disparities in spatial access to health care facilities, and late-stage breast cancer diagnosis in metropolitan Detroit. Health Place. (2010) 16:1038–52. doi: 10.1016/j.healthplace.2010.06.012, PMID: 20630792

[23] Poulson MR Beaulieu-Jones BR Kenzik KM Dechert TA Ko NY Sachs TE. Residential racial segregation and disparities in breast Cancer presentation, treatment, and survival. Ann Surg. (2021) 273:3–9. doi: 10.1097/SLA.0000000000004451, PMID: 32889878

[24] Mobley LR Kuo T-MM Driscoll D Clayton L Anselin L. Heterogeneity in mammography use across the nation: separating evidence of disparities from the disproportionate effects of geography. Int J Health Geogr. (2008) 7:32. doi: 10.1186/1476-072X-7-32, PMID: 18590540 PMC2474591

[25] Haas JS Earle CC Orav JE Brawarsky P Neville BA Williams DR. Racial segregation and disparities in cancer stage for seniors. J Gen Intern Med. (2008) 23:699–705. doi: 10.1007/s11606-008-0545-9, PMID: 18338215 PMC2324162

[26] Moss JL Wang M Liang M Kameni A Stoltzfus KC Onega T. County-level characteristics associated with incidence, late-stage incidence, and mortality from screenable cancers. Cancer Epidemiol. (2021) 75:102033. doi: 10.1016/j.canep.2021.102033, PMID: 34560364 PMC8627446

[27] Warner ET Gomez SL. Impact of neighborhood racial composition and metropolitan residential segregation on disparities in breast cancer stage at diagnosis and survival between black and white women in California. J Community Health. (2010) 35:398–408. doi: 10.1007/s10900-010-9265-2, PMID: 20358266 PMC2906635

[28] Mobley LR Subramanian S Tangka FK Hoover S Wang J Hall IJ. Breast Cancer screening among women with Medicaid, 2006-2008: a multilevel analysis. J Racial Ethn Health Disparities. (2017) 4:446–54. doi: 10.1007/s40615-016-0245-9, PMID: 27287274 PMC5863532

[29] Moss JL Ehrenkranz R Perez LG Hair BY Julian AK. Geographic disparities in cancer screening and fatalism among a nationally representative sample of US adults. J Epidemiol Community Health. (2019) 73:1128–35. doi: 10.1136/jech-2019-212425, PMID: 31615890

[30] Ouédraogo S Dabakuyo-Yonli TS Roussot A Pornet C Sarlin N Lunaud P. European transnational ecological deprivation index and participation in population-based breast cancer screening programmes in France. Prev Med. (2014) 63:103–8. doi: 10.1016/j.ypmed.2013.12.007, PMID: 24345603

[31] Pornet C Dejardin O Morlais F Bouvier V Launoy G. Socioeconomic and healthcare supply statistical determinants of compliance to mammography screening programs: a multilevel analysis in Calvados. France Cancer Epidemiol. (2010) 34:309–15. doi: 10.1016/j.canep.2010.03.010, PMID: 20403737

[32] Deborde T Chatignoux E Quintin C Beltzer N Hamers FF Rogel A. Breast cancer screening programme participation and socioeconomic deprivation in France. Prev Med. (2018) 115:53–60. doi: 10.1016/j.ypmed.2018.08.006, PMID: 30099047

[33] Douglas E Waller J Duffy SW Wardle J. Socioeconomic inequalities in breast and cervical screening coverage in England: are we closing the gap? J Med Screen. (2016) 23:98–103. doi: 10.1177/0969141315600192, PMID: 26377810 PMC4855247

[34] Jack RH Robson T Davies EA. The varying influence of socioeconomic deprivation on breast cancer screening uptake in London. J Public Health Oxf Engl. (2016) 38:330–4. doi: 10.1093/pubmed/fdv038, PMID: 25829530

[35] Jack RH Møller H Robson T Davies EA. Breast cancer screening uptake among women from different ethnic groups in London: a population-based cohort study. BMJ Open. (2014) 4:e005586. doi: 10.1136/bmjopen-2014-005586, PMID: 25324320 PMC4202018

[36] Massat N Douglas E Waller J Wardle J Duffy S. Variation in cervical and breast cancer screening coverage in England: a cross-sectional analysis to characterise districts with atypical behaviour. BMJ Open. (2015) 5:e007735. doi: 10.1136/bmjopen-2015-007735, PMID: 26209119 PMC4521532

[37] Renshaw C Jack RH Dixon S Møller H Davies EA. Estimating attendance for breast cancer screening in ethnic groups in London. BMC Public Health. (2010) 10:157. doi: 10.1186/1471-2458-10-157, PMID: 20334699 PMC2850886

[38] Zidar MN Larm P Tillgren P Akhavan S. Non-attendance of mammographic screening: the roles of age and municipality in a population-based Swedish sample. Int J Equity Health. (2015) 14:157. doi: 10.1186/s12939-015-0291-7, PMID: 26715453 PMC4696103

[39] Sándor J Brantmüllet É Bödecs T Bálint L Szücs M Péntek E. The introduction of call-recall method into national cancer screening program organization and the social gradient of participation. Studia Universitatis babes-Bolyai. Sociologia. (2008) 2:39–62.

[40] Újhelyi M Pukancsik D Kelemen P Kovács E Kenessey I Bak M. Barriers to organized mammography screening programs in Hungary: a questionnaire-based study of 3, 313 women. Anticancer Res. (2018) 38:1727–34. doi: 10.21873/anticanres.12408 PMID: 29491109

[41] Pataki J Dombrádi V Sárváry A Szőllősi GJ. Breast cancer screening and its associating factors among hungarian women aged 45-65: a cross-sectional study based on the European health interview surveys from 2009 to 2019. BMC Public Health. (2023) 23:1679. doi: 10.1186/s12889-023-16608-5, PMID: 37653363 PMC10472565

[42] Ádány R. Roma health is global ill health. Eur J Pub Health. (2014) 24:702–3. doi: 10.1093/eurpub/cku143, PMID: 25239818

[43] Durham University. Health inequalities in the EU: final report of a consortium Consortium lead: Sir Michael Marmot. Luxembourg: Publications Office of the European Union; (2013).

[44] Kasabji F Vincze F Lakatos K Pálinkás A Kőrösi L Ulicska L. Cross-sectional comparison of health care delivery and reimbursement between segregated and nonsegregated communities in Hungary. Front Public Health. (2024) 12:1152555. doi: 10.3389/fpubh.2024.1152555, PMID: 38327575 PMC10847262

[45] Kósa K Daragó L Ádány R. Environmental survey of segregated habitats of Roma in Hungary: a way to be empowering and reliable in minority research. Eur J Pub Health. (2011) 21:463–8. doi: 10.1093/eurpub/ckp097, PMID: 19617380

[46] Samuels SJ Beaumont JJ Breslow NE. Power and detectable risk of seven tests for standardized mortality ratios. Am J Epidemiol. (1991) 133:1191–7. doi: 10.1093/oxfordjournals.aje.a115831, PMID: 2035521

[47] Williams DR Collins C. Racial residential segregation: a fundamental cause of racial disparities in health. Public Health Rep Wash DC. (1974) 2001:404–16. doi: 10.1093/phr/116.5.404 PMC1497358 12042604

[48] Kirby JB Taliaferro G Zuvekas SH. Explaining racial and ethnic disparities in health care. Med Care. (2006) 44:I64–72. doi: 10.1097/01.mlr.0000208195.83749.c3 PMID: 16625066

[49] LaVeist TA. Racial segregation and longevity among African Americans: an individual-level analysis. Health Serv Res. (2003) 38:1719–34. doi: 10.1111/j.1475-6773.2003.00199.x, PMID: 14727794 PMC1360970

[50] Polednak AP. Trends in US urban black infant mortality, by degree of residential segregation. Am J Public Health. (1996) 86:723–6. doi: 10.2105/AJPH.86.5.723, PMID: 8629726 PMC1380483

[51] Çakir M. Evaluation of preventive health practices in Turkey. Iran J Public Health. (2023) 52:315–24. doi: 10.18502/ijph.v52i2.11884, PMID: 37089157 PMC10113584

[52] Wu S Liang D Shi J Li D Liu Y Hao Y. Evaluation of a population-based breast cancer screening in North China. J Cancer Res Clin Oncol. (2023) 149:10119–30. doi: 10.1007/s00432-023-04905-w, PMID: 37266660 PMC10423103

[53] Silva DMD Cavalcante YA Oliveira BLCA Lopes MVO Fernandes AFC Pinheiro AKB. Social health determinants associated with mammography performance according to the 2013 and 2019 National Health Survey. Ciênc Saúde Colet. (2025) 30:e11452023. doi: 10.1590/1413-81232025301.11452023, PMID: 39879462

[54] Seyedkanani E Hosseinzadeh M Mirghafourvand M Sheikhnezhad L. Breast cancer screening patterns and associated factors in Iranian women over 40 years. Sci Rep. (2024) 14:15274. doi: 10.1038/s41598-024-66342-0, PMID: 38961238 PMC11222508

[55] Omisore AD Odedeyi AA Famurewa OC Olasehinde O Olugbade OT Esan OT. Practice, perceptions, and prospects of mammography screening in Nigeria: insights from a National Survey of female health workers. Clin Breast Cancer. (2022) 22:462–72. doi: 10.1016/j.clbc.2022.02.009, PMID: 35305929

[56] Breast cancer screening differs among Member States. Available online at:https://ec.europa.eu/eurostat/web/products-eurostat-news/-/ddn-20200109-1. Accessed 17 May 2024.

[57] Beating Cancer Inequalities in the EU: Spotlight on Cancer Prevention and Early Detection. Available online at:https://www.oecd-ilibrary.org/social-issues-migration-health/beating-cancer-inequalities-in-the-eu_14fdc89a-en. Accessed 17 May 2024.

[58] Lau J Shrestha P Shaina Ng J Jianlin Wong G Legido-Quigley H Tan K-K. Qualitative factors influencing breast and cervical cancer screening in women: a scoping review. Prev Med Rep. (2022) 27:101816. doi: 10.1016/j.pmedr.2022.101816, PMID: 35656228 PMC9152777

[59] Chorley AJ Marlow LAV Forster AS Haddrell JB Waller J. Experiences of cervical screening and barriers to participation in the context of an organised programme: a systematic review and thematic synthesis. Psychooncology. (2017) 26:161–72. doi: 10.1002/pon.4126, PMID: 27072589 PMC5324630

[60] Teo CH Ng CJ Booth A White A. Barriers and facilitators to health screening in men: a systematic review. Soc Sci Med. (1982) 165:168–76. doi: 10.1016/j.socscimed.2016.07.023, PMID: 27511617

[61] Mouslim MC Johnson RM Dean LT. Healthcare system distrust and the breast cancer continuum of care. Breast Cancer Res Treat. (2020) 180:33–44. doi: 10.1007/s10549-020-05538-0, PMID: 31983018 PMC7675785

[62] Willems B Bracke P. Participants, physicians or Programmes: participants’ educational level and initiative in cancer screening. Health Policy Amst Neth. (2018) 122:422–30. doi: 10.1016/j.healthpol.2018.02.001, PMID: 29454541

[63] Shah SC Kayamba V Peek RM Heimburger D. Cancer control in low-and middle-income countries: is it time to consider screening? J Glob Oncol. (2019) 5:1–8. doi: 10.1200/JGO.18.00200, PMID: 30908147 PMC6452918

[64] Masi CM Blackman DJ Peek ME. Interventions to enhance breast cancer screening, diagnosis, and treatment among racial and ethnic minority women. Med Care Res Rev MCRR. (2007) 64:195S–242S. doi: 10.1177/1077558707305410, PMID: 17881627 PMC2657605

[65] Colleoni M Bartholomew T Schmidt H. Financial incentives in breast cancer screening: the urgent need to shift from incentivising uptake to promoting active, informed choice through the provision of evidence-based decision aids. BMJ Evid-Based Med. (2022) 27:A6–6. doi: 10.1136/bmj-2021-065726

[66] Bongaerts THG Büchner FL Nierkens V Crone MR Guicherit OR Numans ME. Perceptions and beliefs of general practitioners on their role in the cancer screening programmes in the Netherlands: a mixed-methods study. BMC Prim Care. (2024) 25:129. doi: 10.1186/s12875-024-02394-5, PMID: 38658815 PMC11040810

