# Supplementary material for: Breast cancer screening coverage is severely reduced among women who reside in segregated areas: a cross-sectional investigation in Hungary
Source: Front Public Health. 2025 Jul 14;13:1500098. doi: 10.3389/fpubh.2025.1500098 (PMC12301353; doi:10.3389/fpubh.2025.1500098)

Appendix

Table A1 Proportion (with 95% confidence interval) of GMPs with significantly lower breast cancer screening coverage in segregations compared with that in non-segregated population by Hungarian counties.

Counties	Number of GMPs with relative coverage in SA			Total number of GMPs	Proportion of GMPs with significantly lower coverage in SA than in CA
	Significantly lower	Not deviated from the CA	Significantly higher		
Budapest	4	705	0	709	0.56% [0.15 - 1.44%]
Baranya	4	171	0	175	2.29% [0.63 - 5.75%]
Bács-Kiskun	5	176	0	181	2.76% [0.9 - 6.33%]
Békés	5	127	0	132	3.79% [1.24 - 8.62%]
Borsod-Abaúj-Zemplén	59	276	1	336	17.56% [13.64 - 22.06%]
Csongrád-Csanád	0	86	0	86	0% [0 - 4.2%]
Fejér megye	1	112	0	113	0.88% [0.02 - 4.83%]
Győr-Moson-Sopron	0	100	0	100	0% [0 - 3.62%]
Hajdú-Bihar	19	178	1	198	9.6% [5.88 - 14.58%]
Heves	13	122	0	135	9.63% [5.23 - 15.9%]
Komárom-Esztergom	0	86	0	86	0% [0 - 4.2%]
Nógrád	10	84	0	94	10.64% [5.22 - 18.7%]
Pest	6	348	0	354	1.69% [0.62 - 3.65%]
Somogy	2	130	0	132	1.52% [0.18 - 5.37%]
Szabolcs-Szatmár-Bereg	44	209	1	254	17.32% [12.88 - 22.55%]
Jász-Nagykun-Szolnok	11	159	0	170	6.47% [3.27 - 11.28%]
Tolna	4	86	0	90	4.44% [1.22 - 10.99%]
Vas	0	52	0	52	0% [0 - 6.85%]
Veszprém	1	85	1	87	1.15% [0.03 - 6.24%]
Zala	2	71	0	73	2.74% [0.33 - 9.55%]
Végösszeg	190	3363	4	3557	5.34% [4.63 - 6.13%]

Figure A1 Proportion (and 95% confidence interval) of GMPs with significantly lower breast cancer screening coverage in segregations compared with that in non-segregated population in the Hungarian counties.

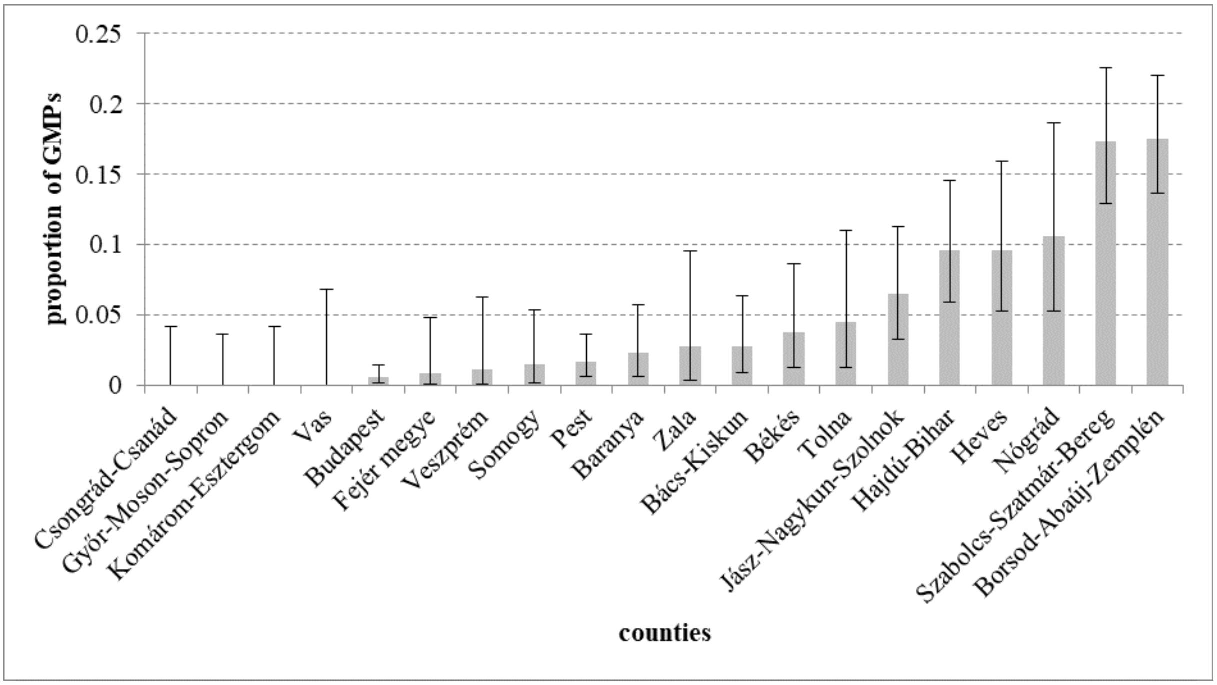

Supplement: Supplementary file 1 [file Data_Sheet_1.pdf]
